# PCR-positive meningococcal CSF infection without pleocytosis but high IL-6 and IL-8

**DOI:** 10.1007/s15010-024-02275-0

**Published:** 2024-04-27

**Authors:** Susanne Dyckhoff-Shen, Hans-Walter Pfister, Uwe Koedel, Matthias Klein

**Affiliations:** 1 Department of Neurology with Friedrich-Baur-Institute, LMU University Hospital, LMU Munich, Marchioninistr. 15, 81377 Munich, Germany; 2 Emergency Department, LMU University Hospital, LMU Munich, Marchioninistr. 15, 81377 Munich, Germany

**Keywords:** Meningococcal meningitis, Sepsis, *Neisseria meningitidis*, Cerebrospinal fluid

## Abstract

**Background:**

Meningococcal meningitis is still a severe disease causing high mortality and morbidity rates. Early diagnosis is crucial to ensure prompt antibiotic therapy. However, identification of the pathogen can be challenging.

**Case presentation:**

A 32-year-old male patient with systemic lupus erythematosus (SLE) presented to the emergency room with fever, nausea, vomiting, headache and lower back pain as well as multiple petechial bleedings. On suspicion of meningococcal infection, the emergency doctor had already administered one dose of ceftriaxone before arrival to the clinic. Blood works showed massive inflammation due to bacterial infection. Cerebrospinal fluid (CSF) analysis showed normal cell count, protein and glucose levels but PCR was positive for *Neisseria meningitis* and IL-6 as well as IL-8 were elevated. On antibiotic therapy with ceftriaxone, the patient’s condition improved quickly.

**Conclusions:**

We present a rare case of meningococcal infection of the CSF in a SLE patient without further CSF abnormalities. We discuss the involvement of early antibiotic treatment and the role of the patient’s immune status in the normal CSF findings of this case. Moreover, this case demonstrates the importance of early antibiotic therapy in bacterial meningitis for the clinical outcome.

## Background

Bacterial meningitis still poses a severe health threat worldwide, with incidence rates ranging from 0.9 to 80 per 100,000 persons and a mortality rate of up to 54% in low-income countries [1]. *Neisseria *(*N.*)* meningitidis* is the second most common causative pathogen of bacterial meningitis in Western countries, causing 11–33% of all cases [2]. In European countries, fatality rate has declined to 4% and unfavorable outcome to 16% in meningococcal meningitis over the last years [3]. *N. meningitidis* enters typically via colonization of the nasopharynx by droplet infection. In 10–20%, the bacteria enter the bloodstream after an incubation period of 1–10 days. If the immune system is unable to eliminate the meningococci, they can invade the meninges or other local sites leading to meningitis [4]. Typical symptoms of meningitis are headache, neck stiffness, and fever; in 2/3 of the patients with meningococcal meningitis, a (petechial) rash can be found [3]. Yet, cardinal symptoms of bacterial meningitis, which include altered consciousness, fever, and nuchal rigidity, may be notably absent in over half of the patients, particularly in those who are immunocompromised impeding the diagnostic process [5]. Overall, the diagnosis can only be established or ruled out through the analysis of the CSF where a pleocytosis, elevated protein > 1 g/l and low glucose are common findings and indicators for bacterial meningitis, yet without standardized cut-off values.

## Case presentation

**Fig. 1:**
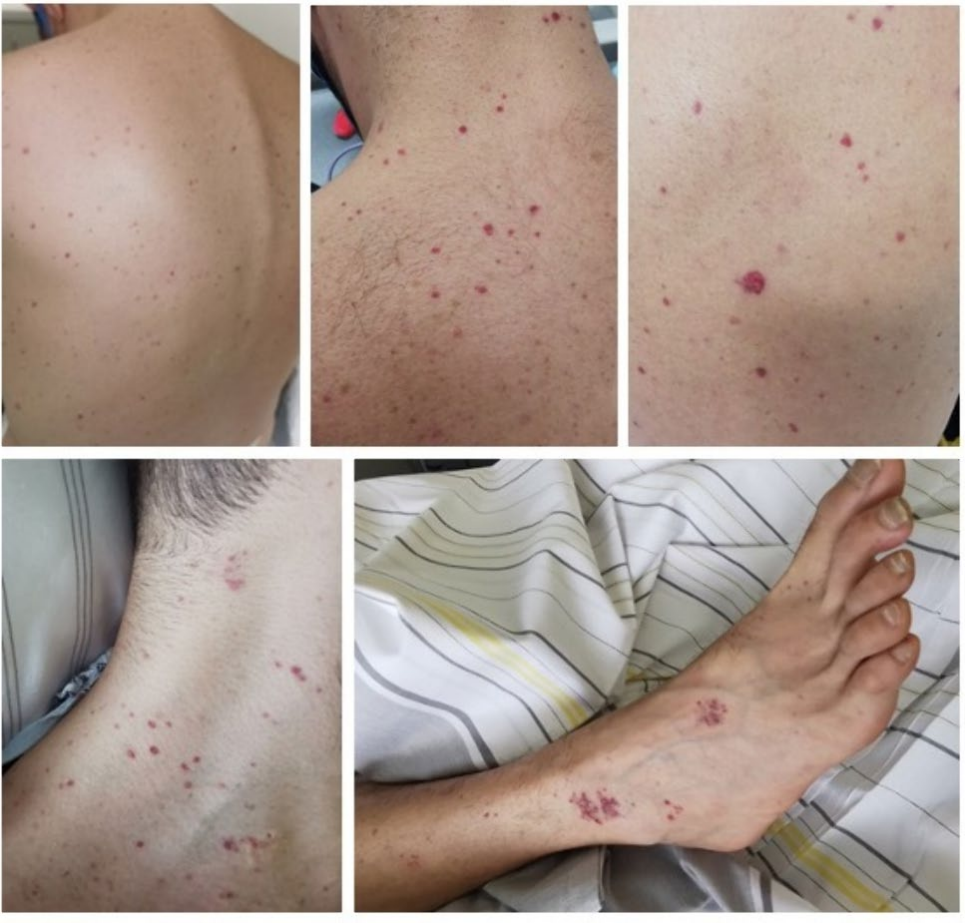
Petechial rash in our patient with meningococcal infection

A 32-year-old Caucasian male with history of systemic lupus erythematosus (SLE), ileostomy, end-stage renal disease and immune-modulating treatment with hydroxychloroquine called the ambulance because he suffered from fever, nausea, vomiting, headache and lower back pain since the previous day. As a rash was noted, the emergency doctor administered 2 g ceftriaxone as he suspected meningococcal disease and transported the patient to the hospital. At the time of admission, vital parameters were normal oxygen saturation (97%), elevated heart rate (133 bpm) and blood pressure (148/100 mmHg) as well as fever (39.1 °C). Moreover, multiple petechial bleedings on both arms, legs and trunk were visible (Fig. 1). Upon neurologic examination, the patient was alert, oriented and showed neck stiffness, but no focal neurological deficit. On admission, the patient’s blood works showed leukocytosis of 16.9 G/l, C-reactive protein (CRP) of 13.6 mg/dl (reference value: ≤ 0.5 mg/dl), procalcitonin (PCT) of > 100 ng/ml (reference value: ≤ 0.1 ng/ml) and interleukin-6 (IL-6) of 41,902 pg/ml (reference value: ≤ 5.9 pg/ml), indicating a severe bacterial infection. Interestingly, the results of an immediate lumbar puncture showed a normal CSF cell count (3 cells/µl), normal erythrocyte count (4/µl), normal CSF protein level (21 mg/dl) and normal CSF glucose (59 mg/dl, serum glucose: 91 mg/dl, CSF/blood index: 0.65). Next, the patient’s CSF underwent microbiologic assessment: multiplex PCR was positive for *N. meningitidis* while CSF culture remained negative. *N. meningitidis* antigen from serum was negative. Initial blood cultures were not available.

As sepsis was suspected, the patient was admitted to our neurological intensive care unit for monitoring and treatment of potential complications. He received antibiotic therapy with ceftriaxone as well as fluids and analgesic medication. Imaging of thorax and abdomen revealed no infectious focus. A cranial MRI scan revealed a few periventricular and subcortical white matter lesions, state after partial mastoidectomy on the left side and a linear shaped defect in the left cerebellar hemisphere, but no restriction of diffusion, no signs of hemorrhage, empyema, abscess or encephalitis.

Under antibiotic therapy, the patient's blood markers of infection improved dramatically within days and the patient could be transferred to the general neurological ward after 3 days on ICU. At day 5, the petechiae disappeared, only slight occasional back pain remained. The patient was discharged after 11 days of ceftriaxone therapy in his previous health status without any neurological deficits. Although a second lumbar puncture to detect a delayed rise of leukocytes in the CSF would have been interesting from a diagnostic point of view, it was not performed due to the risk–benefit analysis, as it would not have had any therapeutic consequences due to the rapid improvement in the clinical condition of the patient.

To unravel if signs of infection were present in the CSF despite the normocellular CSF, we examined the levels of IL-6 and -8 within the patient’s CSF using ELISA (R&D Systems, according to the manufacturer’s instructions, no reference values for CSF available). Both parameters were highly increased (IL-6: 374 pg/ml, IL-8: > 2000 pg/ml) compared to the mean of CSF controls of 8 patients without infection of the CNS (IL-6: 0.64 pg/ml, IL-8: 160 pg/ml). These findings indicate an inflammatory reaction within the CSF on cytokine level in the context of meningococcal meningitis despite lack of pleocytosis and normal protein level.

## Discussion

We report a rare case of an immunocompromised SLE patient with clinical signs of meningitis, CSF PCR positive for *N. meningitidis* but normal CSF cell count, protein and glucose levels. Furthermore, CSF culture remained negative. As a sign of CSF infection, IL-6 and IL-8 were highly elevated in the CSF.

From a clinical point of view, the patient presented with typical signs of meningococcal meningitis such as headache, neck stiffness and fever as well as petechia which are another hallmark of meningococcal infection at first identified by the emergency doctor. The diagnosis of bacterial meningitis is established by investigation of the CSF where pleocytosis, elevated protein and low glucose are typically found. Detection of the pathogen from the CSF, e.g. by bacterial culture proves the diagnosis. Previous studies have shown that the yield of CSF culture is reduced significantly in pretreated patients with meningococcal meningitis [6]. Thus, CSF culture negativity in our case was not unexpected. Moreover, a large cohort study discovered that in pretreated meningococcal patients—contrary to other pathogens—also positive blood cultures, pleocytosis in CSF and positive cultures from other sites were less frequent than in non-pretreated cases [6]. Regarding differences between CSF culture and PCR, a study identified qPCR as over 3 times more sensitive than culture in detection of invasive meningococcal disease [7]. Other studies from the United Kingdom and Spain also showed that 57% and 24%, respectively, of cases with meningococcal disease were confirmed by PCR only [8, 9]. These cases clearly demonstrate the advantage of using multiplex PCR, which can deliver results rapidly, offers a high diagnostic accuracy and allowed us to confirm the diagnosis. The only drawback of PCR compared to CSF culture consists in the fact that it does not offer knowledge on bacterial susceptibility to antibiotics.

A study with 121 patients found highly increased IL-6 levels within the CSF in patients with bacterial and viral meningitis during the acute phase of meningitis [10]. Furthermore, a meta-analysis about the diagnostic performance of interleukin-6 and interleukin-8 for bacterial meningitis reported a sensitivity of 91% and specificity of 93% for IL-6 as well as a sensitivity of 95% and specificity of 89% for IL-8 [11]. Therefore, highly elevated IL-6 and IL-8 levels in our patient’s CSF support his condition of meningitis. A mere contamination of meningococci into the CSF can therefore be ruled out in our patient. Alternative reasons for normal CSF parameters might be an examination at an early state of the disease where the cell count was not yet increased, or the immune status of our patient.

In search of literature, few similar cases have been reported in the past: A prospective study between 1987 and 1990 in Barcelona revealed 82 patients with meningococcal meningitis, 8 of whom showed normal CSF cell count (defined by the cited article as < 10 cells/μl) [12]. Of these 8 patients, 7 were children with mean age of 5.6 years (CSF cell counts: 0–8 cells/µl, reference value: < 10 cells/µl) and one was a 69-year-old man with history of diabetes mellitus (CSF cell count: 3 cells/µl, reference value: < 5 cells/µl). Moreover, from 2 patients with meningococcal meningitis and normal CSF cell count reported by Lessing et al., one was a 5-month-old child and the other a 18-year-old male pretreated with benzylpenicillin prior lumbar puncture [13]. A similar example of a 21-year-old male patient with fulminant meningococcal sepsis, typical meningeal signs, positive CSF and blood cultures for *N. meningitidis*, but normal CSF cell count, protein and glucose levels was reported who displayed a less favorable clinical course than our patient [14]. Typically, meningococcal meningitis patients with normal CSF cell count and chemistry show a high bacterial density in the CSF and a fulminant course of the disease. It can be assumed that the very early treatment of our patient, however, efficiently eradicated bacteria and thus prevented an unfavorable outcome.

A systematic review of cases of bacterial meningitis without CSF pleocytosis identified 99 cases, of which 25 were caused by *N. meningitidis*, and thereof 11 cases in adults [15]. In these 11 cases, 3 displayed elevated CSF protein (cut-off: 45 mg/dl) and none showed abnormal CSF glucose or CSF/blood glucose index. Although none of those 11 patients had comorbidities, death was reported in 2 of them. Most cases of meningitis which initially lack an elevation of CSF cell count showed CSF pleocytosis on second lumbar puncture, suggesting that CSF was taken too early in the course of infection to detect meningeal inflammation [15]. This might be also the reason for normal CSF parameters in our patient, but a second lumbar puncture was not performed to confirm this hypothesis. Some authors discuss meningococcal contamination of CSF by traumatic lumbar puncture or lack of white blood cells in CSF due to leukopenia [12], but neither was the case in our patient as blood leukocytes were rather elevated (16.9 G/l) and erythrocyte count in CSF was 4 per µl, rendering contamination very unlikely.

Notably, meningococcal meningitis in our patient was diagnosed clinically by the emergency physician due to a meningitic syndrome combined with noticeable petechiae. A rapid administration of ceftriaxone in the preclinical setting is rarely reported, but may have contributed to the favorable course and clinical outcome of our patient.

Patients with systemic lupus erythematosus are more susceptible to bacterial and opportunistic infections on one side due to low circulating complement in this immune-complex disease and on the other side due to immunosuppressive treatment [16]. Hung et al. identified 17 SLE patients with CNS infections during a 20-year period: 10 suffered from cryptococcal meningitis, 7 from bacterial meningitis, with an overall mortality rate of 41.2% [17]. Another review identified patients with complement system deficiencies to have a strongly increased risk of meningococcal meningitis [18]. Mitchell et al. observed two cases of meningococcal infections in SLE patients, both under immunosuppressive therapy with corticosteroids [16]. Interestingly, our patient only received hydroxychloroquine as immune-modulating SLE treatment without any corticosteroids or other immunosuppressive medication and additionally, he showed a good clinical outcome despite the poor prognosis of the disease, which is especially the case in SLE patients. While it is already known that a strong host immune reaction contributes to a fatal clinical course and outcome in bacterial meningitis, the role of immune modulating drugs like hydroxychloroquine in meningococcal meningitis remains largely unexplored. Intriguingly, hydroxychloroquine and chloroquine are endosomal Toll-like receptor (TLR) inhibitors—shown to be beneficial in murine pneumococcal meningitis [19]. Endosomal TLR9 was reported to play an important part in immune cell activation upon exposure to *N. meningitidis* [4] suggesting a potentially beneficial effect of hydroxychloroquine in meningococcal meningitis.

This case report highlights several noteworthy aspects in a patient presenting with meningococcal meningitis: the prehospital administration of antibiotics, the unusually normal cerebrospinal fluid parameters despite the detection of *N. meningitidis* via PCR, the favorable clinical course, all while under immunomodulatory therapy due to systemic lupus erythematosus.

One limitation of this case report is that initial blood cultures were not available. Thus, we cannot comment on systemic effects of the preclinical antibiotic treatment on swift bacterial clearance in the blood stream. Furthermore, a second CSF examination was not undertaken and it therefore remains unclear if pleocytosis was present later during the disease.

## Conclusion

This article reports a rare case of a 32-year-old SLE patient with meningococcal meningitis with normal CSF cell count, protein and glucose. Therefore, as seen in other similar cases, normal CSF parameters cannot completely rule out bacterial meningitis suggesting that microbiologic testing (e.g. CSF culture, PCR) should be performed in all cases of suspected meningitis. Moreover, the patient’s positive outcome underlines the clinical importance of prompt antibiotic treatment in every case of suspected bacterial meningitis to improve the patient’s prognosis.

## Data Availability

The data that support the findings of this study are available from the corresponding author upon reasonable request.
